# Identification and validation of candidate biomarkers associated with mitochondria and 18 types of programmed cell death in trigeminal neuralgia: insights from transcriptome sequencing

**DOI:** 10.3389/fnmol.2026.1798469

**Published:** 2026-06-24

**Authors:** Liqin Gao, Hongchen Shi, Lulu Xi, Xiaohui Liu, Yue Ren, Qiujun Wang

**Affiliations:** 1 Department of Pain, The Second Hospital of Hebei Medical University, Shijiazhuang, China; 2 Department of Anesthesiology, Hebei Medical University Third Hospital, Shijiazhuang, China

**Keywords:** machine learning, mitochondria, nomogram, programmed cell death, trigeminal neuralgia

## Abstract

**Background:**

Trigeminal neuralgia (TN) is relatively prevalent among the elderly population. Mitochondria-regulated programmed cell death has been identified as an important mechanism in the pathogenesis of a series of diseases; however, its role in TN remains poorly understood. This study aimed to uncover mitochondria-related genes (MRGs) and programmed cell death-related genes (PCDRGs) implicated in TN and elucidate their potential mechanisms.

**Methods:**

The transcriptomic data of TN were obtained from clinical datasets, while MRGs and PCDRGs were retrieved from published literature. The processed transcriptome data were subjected to differential expression analysis and intersected with MRGs and PCDRGs to identify candidate genes. Subsequently, candidate biomarkers were further screened out via machine learning, and a nomogram was constructed. Additional analyses mainly included protein structure prediction, functional enrichment analysis, regulatory network analysis, drug prediction, and immune microenvironment analysis.

**Results:**

Among the 10 candidate genes, two candidate biomarkers (BCL2A1 and DBT) were employed to build a nomogram that exhibits favorable predictive performance. The protein structures of these biomarkers were predicted using the Human Protein Atlas (HPA) database, with a high level of confidence. Functional enrichment analysis revealed that these two candidate biomarkers might play a critical role in the progression of TN by regulating pathways such as “insulin signaling” and “Fc gamma R-mediated phagocytosis.”

**Conclusion:**

This study identified two candidate biomarkers (BCL2A1 and DBT), which are linked to mitochondria and programmed cell death in TN, and explored their application value in clinical identification, offering novel research orientations for the diagnosis and treatment of TN.

## 1 Introduction

The typical symptom of trigeminal neuralgia (TN) is paroxysmal severe pain in the distribution area of the unilateral trigeminal nerve, which is often described by patients as a pricking, electric shock-like or tearing sensation, and are localized to the distribution area of one or more divisions within the trigeminal territory (Cruccu et al., 2016; Headache Classification Committee of the International Headache Society [IHS], 2013). The incidence rate is estimated to be 5.5 per 100,000 individuals and increases with age (Svedung Wettervik et al., 2023), with a higher prevalence in women than in men (De Stefano et al., 2023). The onset of TN is often triggered by minor facial stimuli, severely restricting daily activities. Simultaneously, prolonged intense pain causes psychological distress, significantly reducing patients’ quality of life (Melek et al., 2019; Wu et al., 2015; Zakrzewska et al., 2017). Carbamazepine and oxcarbazepine are first-line medications, achieving significant pain control in approximately 90% of patients. However, long-term use may lead to diminishing efficacy and adverse effects such as dizziness, diplopia, and ataxia, with about 23% of patients discontinuing treatment due to complications (Shankar Kikkeri and Nagalli, 2025). Interventional treatments for TN include percutaneous radiofrequency thermocoagulation (PRT), percutaneous balloon compression (PBC), and microvascular decompression (MVD). However, previous studies have found that even when patients undergo the best optimal choice–microvascular decompression, recurrence may still occur with sufficiently long-term follow-up (Andersen et al., 2022), relevant studies have shown that based on the 10-years post-operative follow-up data, the recurrence rate of this surgery is 15%–35% (Bezerra et al., 2023). So the management of TN faces significant challenges. The pathogenesis of the disease remains incompletely understood at present. Identifying novel candidate biomarkers is crucial for investigating the pathogenesis of TN, enhancing diagnosis, refining treatments, and developing targeted therapeutics.

Mitochondria serve as a central player in cellular energy generation and the regulation of multiple types of cell death, such as apoptosis and necrosis (Green and Kroemer, 1998; Vakifahmetoglu-Norberg et al., 2017). Given that mitochondrial proteins responsible for activating effector caspases are released, the permeability of the outer mitochondrial membrane serves as a critical regulatory hub in apoptosis, a process controlled by proteins of the Bcl-2 family (Danial and Korsmeyer, 2004).

Any alteration in mitochondrial state can disrupt this mechanism, leading to the accumulation of reactive oxygen species (ROS) and mitochondrial dysfunction, thereby initiating a vicious cycle of cellular damage and death (Zhao et al., 2024). The research discovered severe mitochondrial damage in the injured trigeminal ganglion (TG), spanning transcriptional, translational, and functional impairments, and indicates that enhancing NAD+ levels activates mitochondrial function, improves mitochondrial adaptability, and significantly alleviates trigeminal neuropathic pain (Yang et al., 2025).

Programmed cell death (PCD) is an essential physiological process that contributes to maintaining tissue homeostasis and removing damaged or redundant cells. PCD encompasses 18 distinct mechanisms, including apoptosis, anoikis, autophagy, alkaliptosis, cuproptosis, entosis, entotic cell death, immunogenic cell death, ferroptosis, lysosome-dependent cell death, methuosis, necroptosis, netotic cell death, NETosis, oxeiptosis, pyroptosis, parthanatos, paraptosis (Chen et al., 2024; Qin et al., 2023). In addition, various forms of PCD have been established as critical regulators of neural homeostasis and pathological injury. Different subtypes of programmed cell death (PCD) may play non-redundant and potentially complementary roles in neuropathic pain. Li et al. (2020) reported that inhibiting neuronal apoptosis by upregulating Cdh1 protected GABAergic neurons and alleviated pain; while Cai et al. (2020) demonstrated that modulating astrocytic autophagy could suppress glial activation and reduce pain hypersensitivity. These two distinct mechanisms–targeting neuronal survival and glial inflammation, respectively–suggest that multiple programmed cell death processes may regulate disease progression through different pathways, laying the groundwork for investigating their combined effects in neuropathic pain. Therefore, this study incorporates all 18 PCD types for a panoramic analysis, aiming to systematically characterize the complete regulatory landscape of cell death programs in trigeminal neuralgia, thereby avoiding research bias from focusing on single pathways and enhancing the accuracy and representativeness of biomarker screening. PCD has been identified as a key mediator in the pathogenic mechanisms of various diseases, including autoimmune disorders, cancer, neurodegenerative diseases, immunodeficiencies, and developmental abnormalities (Gibellini and Moro, 2021; Zou et al., 2022). Studies show that the N-terminal domain of pyroptosis executor protein GSDMD (GSDMD-NT) translocates to mitochondria, forming pores in the inner mitochondrial membrane. This process leads to the loss of mitochondrial membrane potential, the generation of ROS, and the release of mitochondrial contents, thereby accelerating and amplifying pyroptosis (Miao et al., 2023). Another study indicates that trigeminal ganglion neurons may exhibit mitochondrial dysfunction, characterized by increased fission and reduced fusion. This results in mitochondrial fragmentation, functional impairment, excessive ROS production, and ultimately oxidative stress-induced neuronal apoptosis (Guan et al., 2025). Bone morphogenetic protein 7 (BMP7) alleviates TN by reducing oligodendrocyte (OL) apoptosis. The mechanism may involve activation of the BMP7-mediated STAT3 and NF-κB/p65 signaling pathways, thereby decreased OL apoptosis (Chen et al., 2023). Both apoptosis and autophagy exert essential functions in cancer, immune processes, and neurodegenerative disorders (Glick et al., 2010; Menzies et al., 2017; Yuan and Yankner, 2000).

Despite extensive research on mitochondrial function and PCD in numerous diseases, their roles in TN remain underexplored. The pathogenesis of TN is still poorly understood, particularly with respect to mitochondrial dysfunction and the lack of biomarkers linked to PCD mechanisms. Therefore, exploring the biomarkers associated with mitochondria and PCD in TN, as well as their molecular mechanisms, is of great significance. In addition, trigeminal neuralgia is a neurological disorder primarily affecting the trigeminal nerve and trigeminal ganglion. Although the pathological core of trigeminal neuralgia lies in the trigeminal ganglion, obtaining trigeminal ganglion tissue from living patients is highly invasive and impractical for routine clinical diagnosis. Peripheral blood, as a source of “liquid biopsy,” contains circulating immune cells that interact with the nervous system during the progression of chronic pain. Therefore, identifying blood-based biomarkers provides a minimally invasive and feasible strategy for the clinical management of trigeminal neuralgia.

This study utilized transcriptome sequencing on 10 pairs of clinical samples to comprehensively explore the predictive associations between mitochondrial dysfunction, PCD, and the pathogenesis of TN. Through bioinformatics approaches including differential expression analysis, functional enrichment analysis, immune infiltration profiling, molecular regulatory network construction, and drug-target prediction, we aimed to provide novel insights for TN clinical management.

## 2 Materials and methods

### 2.1 Sample collection and sequencing

The Second Hospital of Hebei Medical University supplied a total of 10 pairs of frozen whole-blood samples. which included 10 TN-related samples and 10 control samples. All participants filled out and signed an informed consent form, while the ethical review and approval were conducted by the Second Hospital of Hebei Medical University (approval code: 2025-R363). Following collection, these samples were subjected to RNA sequencing (RNA-seq).

The RNA sequencing library was constructed and sequenced by LC Sciences (Hangzhou, China^1^). Total RNA was extracted and then subjected to rigorous quality and purity assessments. mRNA was extracted with Dynabeads Oligo (dT) 25-61005 (Thermo Fisher, CA, USA) before undergoing controlled fragmentation. Reverse transcription was performed on the fragmented mRNA to generate cDNA, employing Invitrogen SuperScript™ II Reverse Transcriptase (Invitrogen, cat. 1896649, USA) as the enzyme. Later, the cDNA library was sequenced on a high-throughput sequencing platform to generate substantial sequence data. The sequencing mode was PE150. Peripheral whole blood was selected as the sequencing sample in this study, mainly owing to its convenient clinical acquisition, non-invasiveness and good reproducibility, which rendered it more suitable for the screening and translational application of clinical biomarkers. The TG, as the core tissue of pathological injury, was difficult to obtain routinely in clinical settings and was therefore not included in this study.

### 2.2 Data processing

To eliminate technical and systematic differences and improve data quality, FastQC and FastP functions in R language (v 4.2.2) (Lopez et al., 2025) were employed to conduct quality control on the raw sequencing reads. Subsequently, alignment of the clean data with the human genome (GRCh38) was carried out by applying the TopHat 2.0 program, and the normalization of Mapped Reads numbers and transcript lengths in the samples was conducted using the FPKM algorithm from StringTie (Pertea et al., 2016; Supplementary Table 1). Afterward, a boxplot was generated to visualize the normalization outcomes.

The levels of gene expression in samples act as critical indicators to examine both the reliability of the experiment and the appropriateness of sample selection. To examine the correlations between the control group and TN group samples in the transcriptome sequencing data, Spearman analysis was executed using the “cor” function in the R package (v 4.2.2), with |correlation coefficients (cor)| > 0.3 and *P* < 0.05 as the thresholds. Boxplots were drawn with the “pheatmap” package (v 1.0.12) to present the results visually (Gu and Hübschmann, 2022).

### 2.3 Differentially expressed and functional enrichment analyses

Differentially expressed genes (DEGs) between TN and control samples were derived using the DESeq2 package (v 1.42.0) (Lopez et al., 2025), with |log2 Fold Change (FC)| > 0.5 and *P* < 0.05 serving as the screening standards. The biological roles of DEGs in TN were further explored, with “clusterProfiler” package (v 4.8.3) (Wu et al., 2021) employed for Gene Ontology (GO) (*P* < 0.05) and Kyoto Encyclopedia of Genes and Genomes (KEGG) analyses (*P* < 0.05). Notably, the top 3 GO terms with the largest numbers of enriched genes in different categories [biological process (BP), cellular component (CC), and molecular function (MF)], as well as the top 10 significant KEGG pathways, were visualized by “ggplot2” package (v 3.4.1) (Gustavsson et al., 2022).

### 2.4 Identification of candidate genes

A total of 1,136 mitochondria-related genes (MRGs) (Qin et al., 2023) and 1,548 programmed cell death related genes (PCDRGs) (Qin et al., 2023) were identified and extracted from relevant literature (Supplementary Table 2). The 18 types of PCD and their corresponding gene data sources were detailed in Supplementary Table 3. This study integrated genes from the 18 PCD subtypes for global analysis.

The intersection of MRGs, PCDRGs and DEGs was determined utilizing “ggvenn” package (v 0.1.9) (Chen and Boutros, 2011) for the purpose of detecting genes related to both mitochondria and programmed cell death in bulk RNA data, thereby classifying them as candidate genes. Additionally, the Search Tool for the Retrieval of Interaction Gene/Proteins (STRING) database, available at https://www.string-db.org, was utilized to construct a map of the complex interaction web between candidate genes at the protein level. Protein-protein interaction (PPI) network was meticulously crafted with an interaction score > 0.15 to ensure reliability. Subsequently, the PPI network was graphically represented, leveraging the capabilities of Cytoscape software (v 3.10.2) (Shannon et al., 2003).

### 2.5 Machine learning analysis

The “glmnet” package (v 4.1.4) (Friedman et al., 2010) was applied, utilizing candidate genes, a least absolute shrinkage and selection operator (LASSO) model was constructed. The model’s performance was evaluated using deviance and five-fold cross-validation, resulting in the selection of LASSO genes from bulk RNA data, with lambda.best determined as lambda.min. Meanwhile, the “caret” R package (v 6.0.93) (Hu et al., 2022) was applied to execute the support vector machine-recursive feature elimination (SVM-RFE) algorithm with candidate genes for the analysis of feature importance. To minimize the model’s prediction error, the optimal number of variables was determined via five-fold cross-validation. Later, the gene set that had the lowest error rate was picked out and designated as the SVM-RFE genes. Finally, the ggvenn package (v 0.1.9)^2^ was used to find the overlapping of the LASSO genes and SVM-RFE genes to obtain the biomarkers.

### 2.6 Structural and functional analysis of biomarkers

In this study, the Human Protein Atlas (HPA) database^3^ was applied to anticipate the structures of the biomarkers. Understanding the protein structure of biomarkers is essential for understanding their mechanisms of action within cellular signaling pathways, which in turn facilitates the identification of potential therapeutic targets. Meanwhile, to further investigate the associations and pathways between the biomarkers and co-enriched genes, a co-expression network of the biomarkers was constructed through GeneMANIA^4^.

Gene Set Enrichment Analysis (GSEA) was applied to the TN samples of the transcriptome sequencing data to explore the signaling pathways encompassing biomarkers linked to mitochondria and programmed cell death.

Specifically, TN patients were first categorized into high- and low-expression groups, where the division was based on the median values of biomarker expression. Subsequently, DESeq2 package (v 1.42.0) was used to conduct differential analysis. Differentially expressed genes were screened with adj.P < 0.05 and |log2FC| > 0.05, and sorted in descending order by log2FC values. Following this, GSEA was conducted on each key gene using the clusterProfiler package (v 4.7.1.3), with the standards of |normalized enrichment score (NES)| > 1 and *P* < 0.05. Reference gene set “c2.cp.kegg_medicus.v2023.2.Hs.symbols.gmt” was obtained from the Molecular Signatures Database (MSigDB)^5^. Notably, enrichplot package (v 1.18.3) (Wang et al., 2022) was utilized to illustrate the top five significant pathways enriched for each biomarker. Furthermore, in the TN samples of the transcriptome sequencing data, the cor function was also used to separately analyze, via Spearman’s method, the correlations between each biomarker and the corresponding genes within its top-ranked enriched pathway (with cor > 0.3 and *P* < 0.05 set as the screening criteria).

### 2.7 Regulatory network construction and drug prediction

To delve into the molecular regulatory mechanisms of biomarkers linked to mitochondria and programmed cell death in TN, transcription factors (TFs) targeting these biomarkers were forecasted via the miRNet database^6^. Additionally, Clues for the pharmacological studies of TN were provided by predicting drugs that targeted biomarkers through Drug-Gene Interaction Database (DGIdb)^7^. Ultimately, Cytoscape software (v 3.10.2) (Liu et al., 2020) was applied to generate visualizations of the TF-mRNA and drug-biomarker networks.

### 2.8 Immune infiltration analysis

Within the TN samples of the transcriptome sequencing data, the immune microenvironment of TN and control groups was investigated. Initially, To show the infiltration scores of 64 immune cell types in both groups, the xCELL algorithm in the xCell package (v 1.1.0) (Aran et al., 2017) was applied. The differential immune infiltrating cell types in TN group were recognized via Wilcoxon test (*P* < 0.05). Thereafter, applying the “cor” function, Spearman correlation analysis was carried out to establish correlations–both among differential immune infiltrating cell types and between them and biomarkers–with the criteria of |cor| > 0.3 and *P* < 0.05.

### 2.9 Statistical analysis

Bioinformatic analyses were carried out via R language (v 4.2.2). Moreover, the Wilcoxon test was applied to investigate inter-group differences, with *P* < 0.05 designated as the significance threshold.

## 3 Results

### 3.1 Overview of transcriptome profiles in TN

**FIGURE 1:**
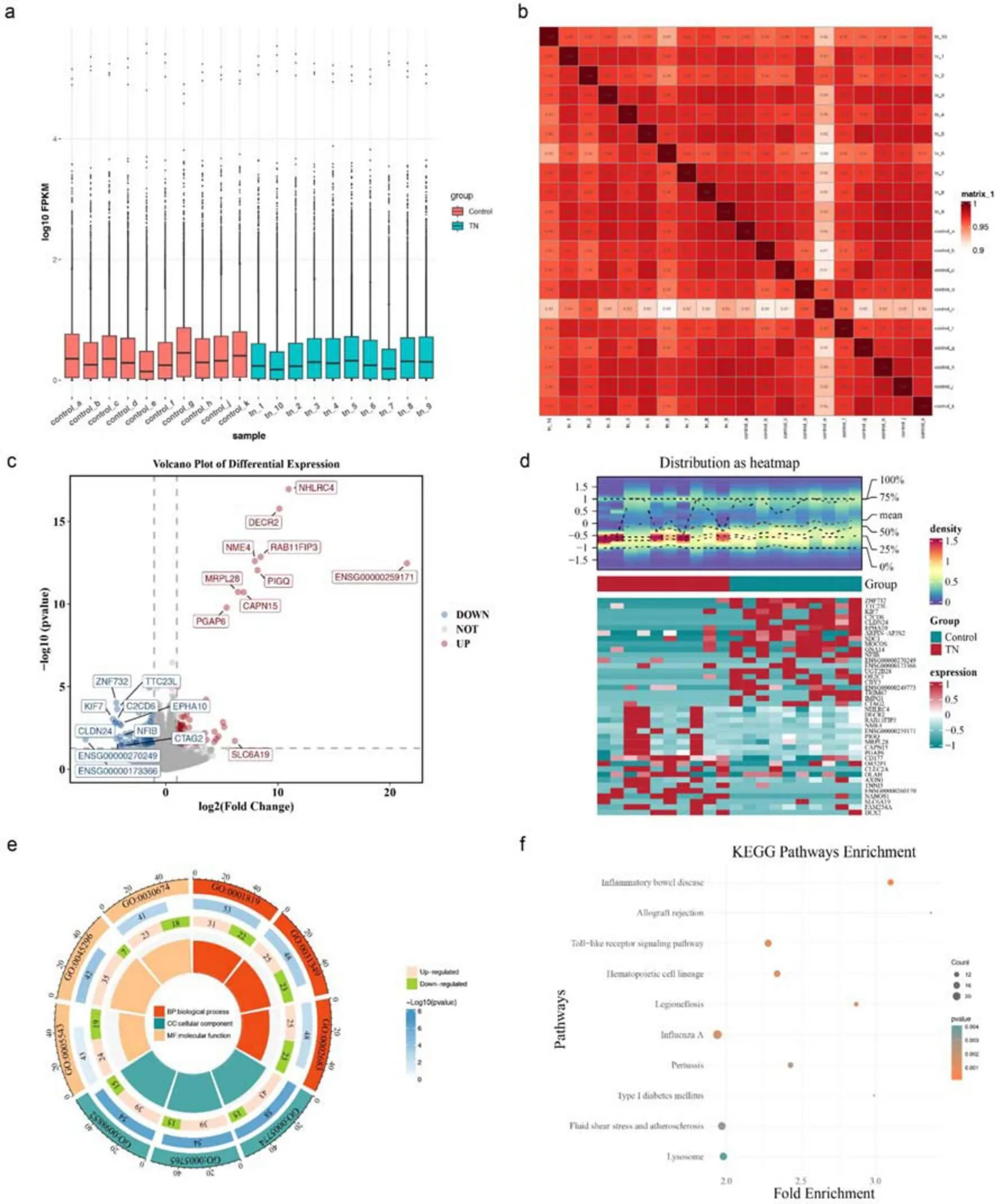
Screening and functions of DEGs. **(a)** FPKM values of each sample in the trigeminal neuralgia (TN) group and control group from transcriptome sequencing data; **(b)** Correlation analysis between the two groups of samples; Volcano plot **(c)** and expression heatmap **(d)** of DEGs between the two groups of samples; Gene Ontology (GO) **(e)** enrichment analysis and Kyoto Encyclopedia of Genes and Genomes (KEGG) **(f)** enriched pathways of DEGs.

After the data normalization processing, it was noted that the overall distribution of gene expression levels in samples from the TN group and the control group was basically aligned, with both showing a distribution that was relatively concentrated (Figure 1a). In addition, the similarity of sample expression patterns was high when comparing the TN group with the control group (|cor| > 0.9, *P* < 0.05) (Figure 1b), which could be used for subsequent research.

After performing differential expression analysis between TN and controls in RNA-seq data, a total of 1,369 DEGs were determined, along with 703 down-regulated and 666 upregulated genes in disease category (*P* < 0.05) (Figures 1c,d and Supplementary Table 4). Among them, ENSG00000259171, ENSG00000270249, and ENSG00000173366 were ENSG numbers and cannot be converted into gene symbols. These genes were considered to be newly discovered in the current database version, or their gene symbols had not been determined yet.

Significantly, 685 GO terms were significantly enriched with DEGs, encompassing 93 MFs, 109 CCs, and 483 BPs (*P* < 0.05) (Supplementary Table 5). For BP, CC, and MF, their respective top 3 enriched pathways comprised “positive regulation of cytokine production,” “lysosomal membrane,” “negative regulation of immune system process,” and “cadherin binding” (Figure 1e). Moreover, The KEGG enrichment analysis performed on the DEGs revealed that 48 pathways were enriched, such as “inflammatory bowel disease,” “allograft rejection,” “apoptosis” and “toll-like receptor signaling pathway” (*P* < 0.05) (Figure 1f and Supplementary Table 5). These extensive associations indicated that the DEGs might have played roles in the pathophysiological process of TN through regulating pathways like the immune response, inflammatory response, and intracellular homeostasis.

### 3.2 The 10 candidate genes were related to each other at the protein level

**FIGURE 2:**
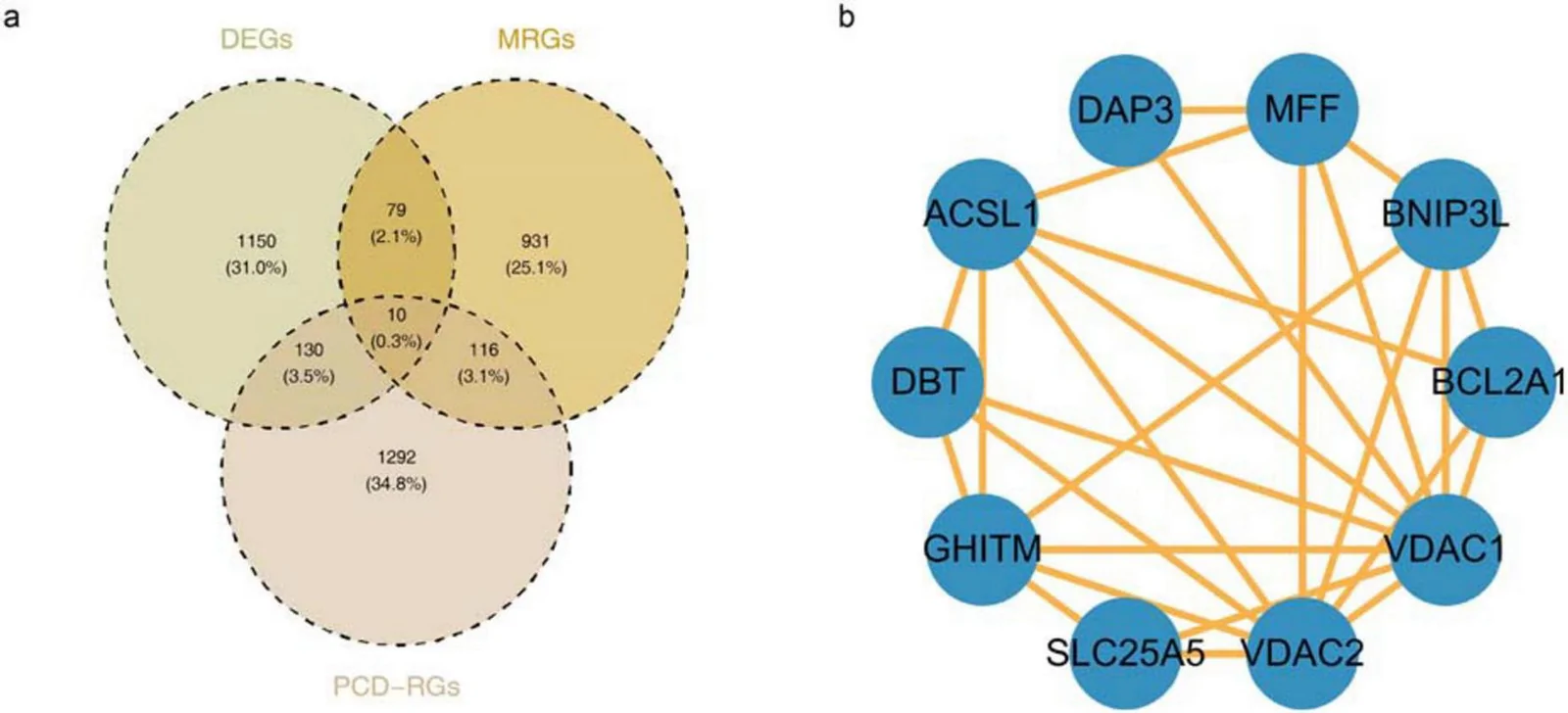
Screening of candidate genes and protein expression. **(a)** Venn diagram for candidate gene screening; **(b)** PPI network of candidate genes. Each small circle represents a candidate gene protein, and the connecting lines indicate the interaction relationships between different proteins.

The intersection of DEGs, MRGs, and PCDRGs served as candidate genes (*n* = 10) for subsequent analytical processes (Figure 2a). In addition, to examine the functional relationships among the 10 candidate genes at the protein level, a PPI network was constructed, revealing 10 interconnected proteins and 26 interaction pairs (Figure 2b). Notably, VDAC2 was found to interact with multiple proteins, including DBT, BCL2A1, and GHITM.

### 3.3 Biomarkers had good predictive values for TN

**FIGURE 3:**
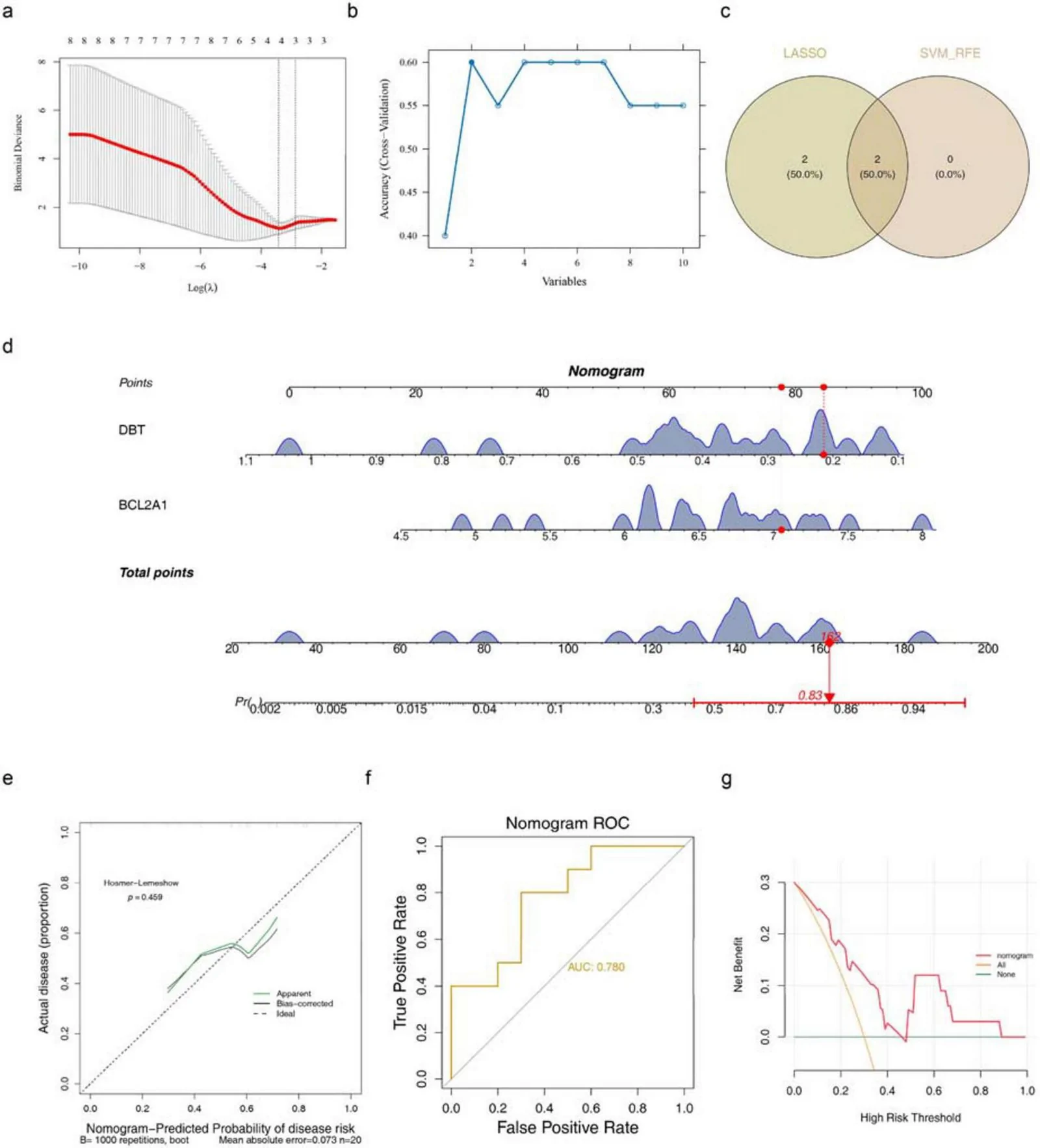
Machine learning screening for biomarkers. **(a)** Coefficient screening plot and cross-validation plot for LASSO analysis; **(b)** Support Vector Machine-Recursive Feature Elimination (SVM-RFE) analysis; **(c)** Venn diagram for biomarker screening; **(d)** Diagnostic nomogram; **(e)** Calibration curve of the nomogram; **(f)** ROC curve of the nomogram model; **(g)** DCA of the nomogram.

When lambda.min = 0.03284711, the LASSO model identified 4 genes associated with TN (Figure 3a). Meanwhile, in the SVM-RFE model, when the number of candidate genes was set to 2, the highest prediction accuracy of the model was achieved (Figure 3b). Following this, the overlapping part of the machine learning results was determined, leading to the identification of 2 biomarkers in this study: BCL2A1 and DBT (Figure 3c).

The nomogram showed that the higher the biomarker scores in the samples, the greater the risk of getting TN (Figure 3d). Additionally, the calibration curve of the nomogram showed *p* = 0.459 and a slope close to 1, which indicated that the nomogram prediction accuracy was excellent (Figure 3e). The Receiver Operating Characteristic (ROC) curve indicated that the nomogram had an AUC of 0.780 (Figure 3f). Meanwhile, the 95% CI of the AUC ranged from 0.55 to 0.95. At the optimal cut-off value of 0.54 for sensitivity and specificity, the sensitivity was 80% and the specificity was 70%. This indicated that when the model-predicted risk was higher than 0.54, 80% of TN patients were correctly identified, and 70% of control subjects were accurately excluded. The effective threshold intervals of the DCA curve were 0–0.46 and 0.49–0.88, and the net benefit of the nomogram model remained above zero. These findings suggested that the model yielded stable positive clinical net benefit within the above threshold ranges and possessed favorable clinical application value (Figure 3g). Furthermore, the GSE186505 dataset was acquired from the Gene Expression Omnibus (GEO) database, and the above nomogram model was reconstructed and validated (Supplementary Figure 1A). Calibration curve analysis showed that the slope of the calibration curve was close to 1 with *p* > 0.05, which indicated good predictive accuracy of the model (Supplementary Figure 1B). DCA revealed that the nomogram curve was consistently superior to the “all” and “none” reference curves (Supplementary Figure 1C). Compared with the extreme strategies of treating all patients or providing no treatment at all, the model generated additional clinical benefits at different threshold probabilities and showed practical application advantages. Collectively, this nomogram model exhibited reliable predictive performance and promising potential value for clinical decision-making.

### 3.4 Crucial functional pathway related to biomarkers

**FIGURE 4:**
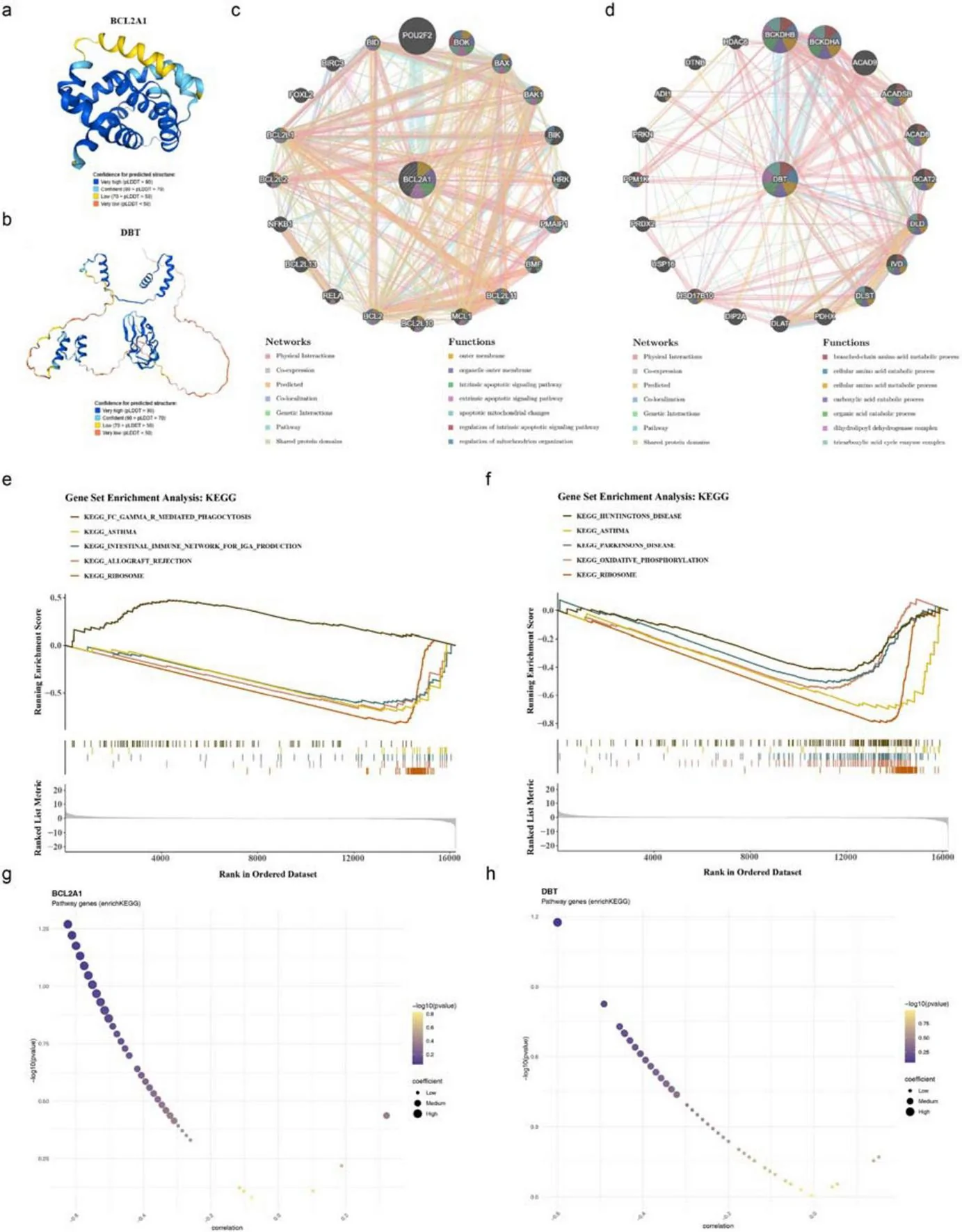
Biomarker-related functions analysis. The structures of biomarker BCL2A1 **(a)** and DBT **(b)** proteins in the HPA database. Different colors represent high confidence, medium confidence, low confidence, and extremely low confidence respectively; Proteins predicted by GeneMANIA to interact with biomarkers BCL2A1 **(c)** and DBT **(d)**; Gene Set Enrichment Analysis (GSEA) of biomarkers BCL2A1 **(e)** and DBT **(f)**; Correlations between biomarkers BCL2A1 **(g)** and DBT **(h)** and genes in TOP1-related pathways.

The structure of the biomarkers was predicted by the HPA database. As shown in Figure 4a, the structure of BCL2A1 was relatively compact and complex, with multiple helical regions and folded parts. Most of the regions were blue, indicating that the predictions for these regions had a high degree of accuracy. In contrast, the structure of DBT was relatively loose. DBT also had a large number of blue regions, but there were also some light blue and yellow parts, which meant that the prediction confidence in these regions was slightly lower than that of BCL2A1 (Figure 4b). Overall, the protein structures of the biomarkers were predicted with a relatively high confidence level based on the HPA database. Subsequently, the predicted structures could be utilized to explore the pathogenesis of the disease from the perspective of protein structures and to design specific drug molecules for this disease.

In GeneMANIA, BCL2A1 was predicted to act synergistically with 20 genes such as BOK, BAX and BCL2L1 through pathways including physical interaction and genetic interaction in the extrinsic apoptotic signaling pathway, organelle outer membrane and intrinsic apoptotic signaling pathway (Figure 4c). Similarly, DBT was predicted to act synergistically with 20 genes like BCKDHB, DLC and BCKDHA participate in physical interactions within pathways like the carboxylic acid catabolic process and the metabolic process of cellular amino acids (Figure 4d).

Through GSEA, it was found that BCL2A1 and DBT were enriched in 19 and 11 pathways, respectively (Supplementary Table 6). The top five significant pathways that each key gene enriched are illustrated in Figures 4e, f. Notably, these genes showed co-enrichment in 6 critical pathways, for example, “insulin signaling” and “Fc gamma R mediated phagocytosis” (*P* < 0.05). In brief, mitochondrial dysfunction and PCD may be potentially involved in TN progression by regulating these pathways. Moreover, most of the 80 genes on the ribosome pathway were negatively correlated with BCL2A1 and DBT (Figures 4g, h). The biomarker genes and the genes in the enriched pathways were likely to be involved in different biological processes within the cell, and there was an antagonistic relationship among these processes.

### 3.5 Drugs and TFs that targeted the biomarkers associated with mitochondria and programmed cell death

**FIGURE 5:**
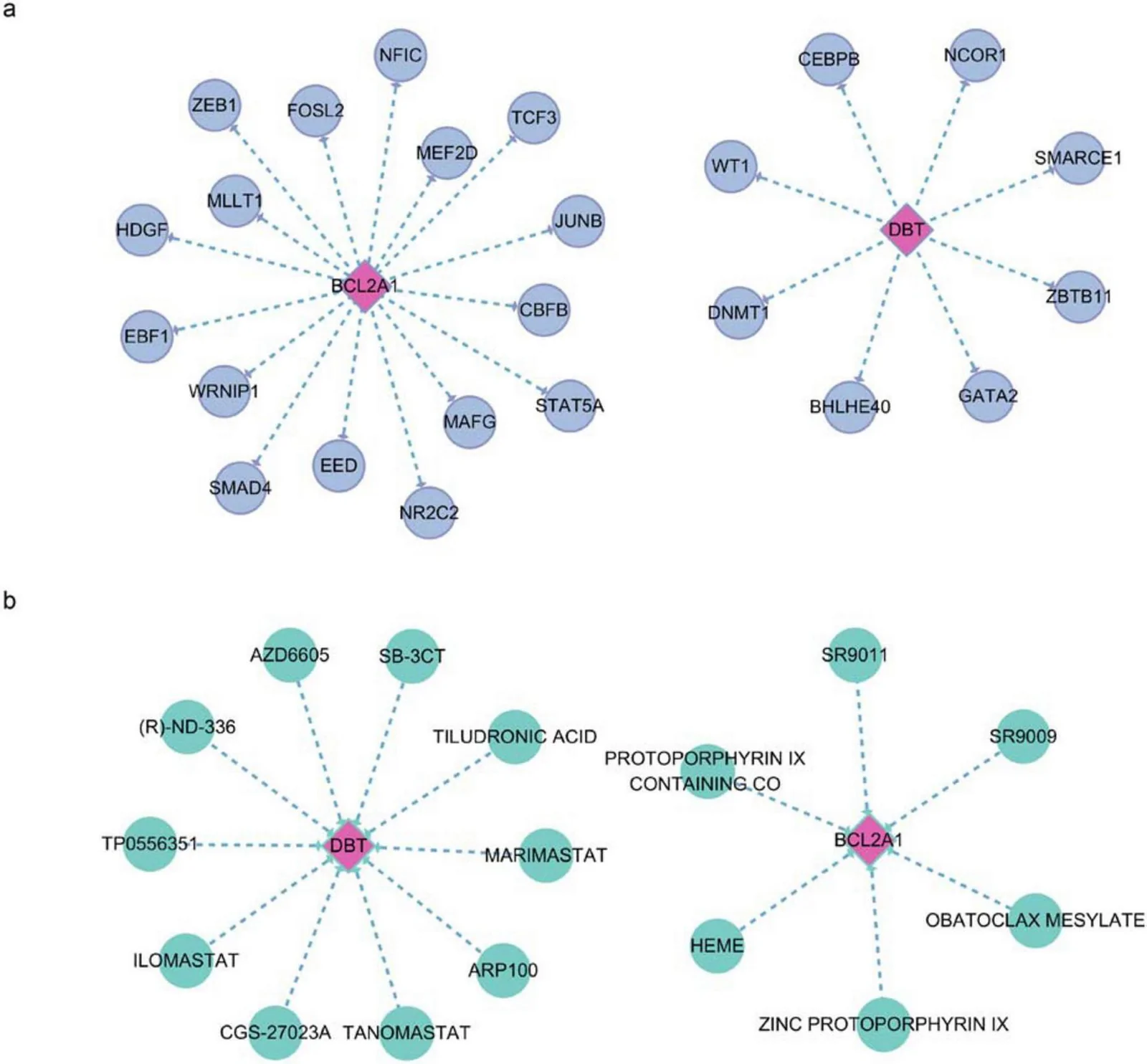
Molecular regulatory networks. **(a)** TFs-biomarker regulatory network, the red part in the middle is the biomarker, and the transcription factors predicted by this biomarker are within the blue circles around it; **(b)** Drug-biomarker interaction network diagram, the red one in the middle is the biomarker, and the green circle around it is the drug predicted by the biomarker.

The TFs-mRNA network showed that a total of 16 transcription factors, such as ZEB1 and MLLT1, were predicted for BCL2A1, and a total of 8 transcription factors, such as NCOR1 and GATA2, were predicted for DBT (Figure 5a). Interestingly, no common transcription factors were predicted for these two biomarkers. This suggested that they might have functioned in different gene regulatory networks, and their transcriptional regulatory mechanisms and the biological processes they were involved in were relatively independent.

Based on the DGIdb database, a total of 6 drugs were predicted for BCL2A1, with 10 drugs in total predicted for DBT (Figure 5b). According to the interaction scores, the top two drugs predicted for BCL2A1 were protoporphyrin ix containing co and zinc protoporphyrin ix, and the top two drugs predicted for dbt were tanomastat and arp100.

### 3.6 Altered immune microenvironment profiles in TN

**FIGURE 6:**
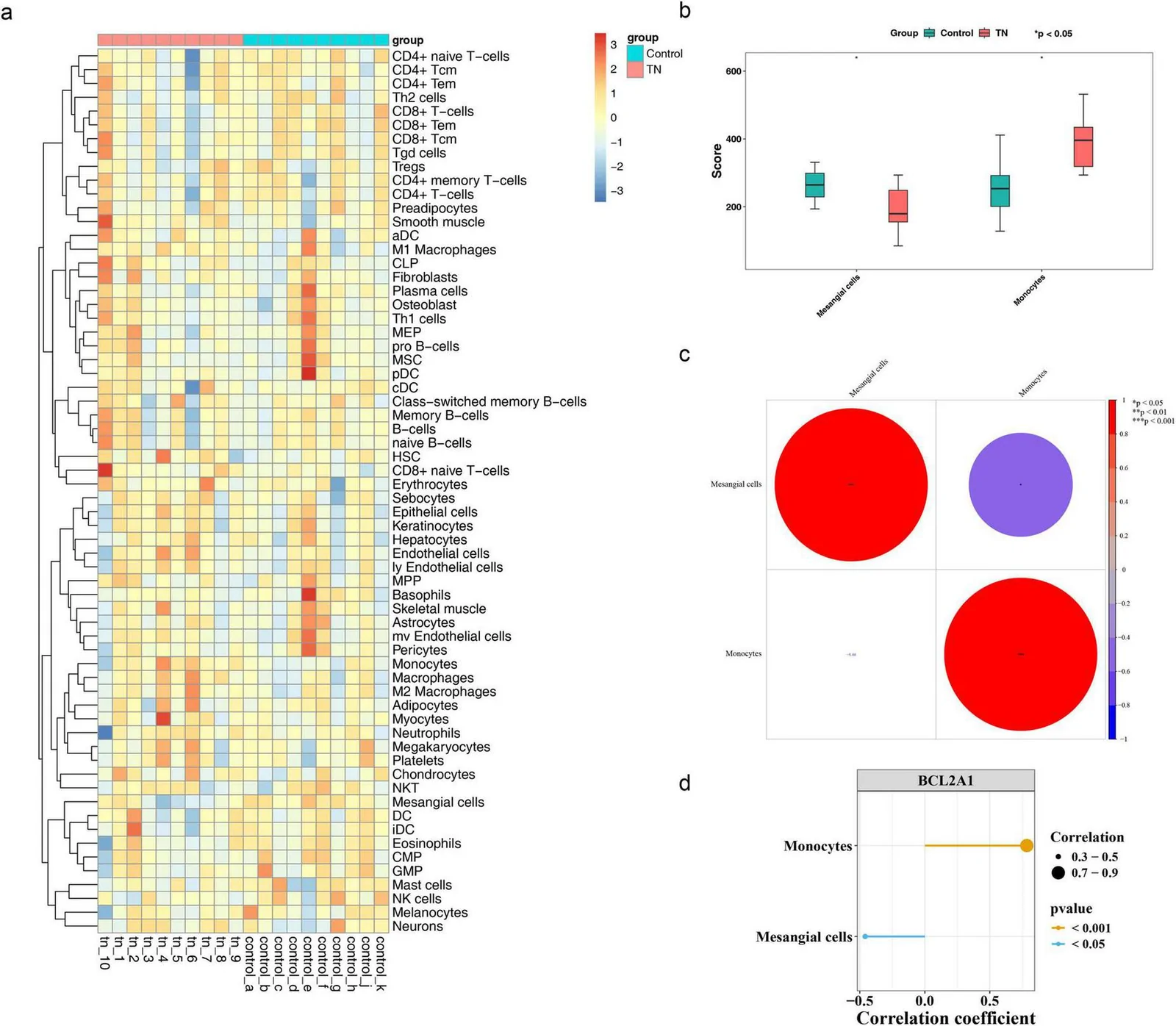
Immune infiltration analysis. **(a)** Heatmap of immune cell infiltration levels in samples from the TN group and control group; **(b)** Boxplot of XCell scores for differentially infiltrating immune cells between groups, *p < 0.05; **(c)** Correlations among immune cells with differential abundance, *p < 0.05, **p < 0.01, ***p < 0.001; **(d)** Correlations between biomarkers and immune cells with differential abundance.

In the transcriptome sequencing data, the immune microenvironment of the TN group and the control group was characterized. Initially, results from observations showed no notable difference in immune cell infiltration levels when comparing the TN group with the control group. However, the control-e immune cells exhibited higher levels of infiltration. There existed differences in immune cell infiltration levels between the TN-10 and TN-6 samples (*P* < 0.05) (Figure 6a). Notably, two differential immune infiltrating cell types were identified in the TN group. Among them, relative to the control group, the TN group had a significantly elevated infiltration level of monocytes, and a significantly reduced infiltration level of mesangial cells (*P* < 0.05) (Figure 6b). Furthermore, there was a notable negative correlation between monocytes and mesangial cells, with the correlation coefficient being −0.46 (*P* < 0.05) (Figure 6c). Similarly, significant correlations were found between BCL2A1 and the differential immune infiltrating cell types (Figure 6d). BCL2A1 correlated positively with monocytes (cor > 0.7, *P* < 0.001) but negatively with mesangial cells (cor < −0.3, *P* < 0.05). In contrast, DBT showed a relatively mild correlation with the differentially infiltrated immune cells, where the absolute value of the correlation coefficient did not exceed 0.3 (|cor| < 0.3), as shown in Table 1.

**TABLE 1:** Correlations between biomarkers DBT and immune cells with differential abundance.

Gene	Cell	Correlation	*P*-value
DBT	Mesangial cells	−0.1082707	0.6496
DBT	Monocytes	−0.1473684	0.5352

## 4 Discussion

Trigeminal neuralgia is a common neuropathic pain disorder that severely impairs patients’ quality of life. Mitochondria not only provide approximately 95% of cellular energy but also participate in critical processes including cellular signal transduction, regulation of apoptosis, and maintenance of intracellular redox potential and electrolyte homeostasis (Reuter, 2022). PCD is essential for sustaining tissue homeostasis and clearing out damaged or unnecessary cells (Qin et al., 2023). Studies indicate that TN may induce neuronal inflammation and apoptosis, which correlates with upregulated CD95/CD95L expression, leading to neurodegeneration. Mitochondrial dysfunction likely contributes to the regulation of apoptosis in this process (Wang et al., 2020). In a TN rat model, Bone Morphogenetic Protein 7 (BMP7) alleviates trigeminal pain by reducing OL apoptosis and demyelination. This highlights the pivotal role of OL apoptosis in PCD during TN pathogenesis, while mitochondrial functional status may influence OL apoptosis (Chen et al., 2023). Against this background, this study utilized transcriptomic datasets and bioinformatics methods to systematically explore the diagnostic value and biological roles of mitochondria and 18 PCD pathways in TN. Two biomarkers, BCL2 related protein A1 (BCL2A1) and dihydrolipoamide branched-chain transacylase E2 (DBT), were identified as potential diagnostic and therapeutic targets, thus offering new perspectives on the mitochondrial- and PCD-related pathogenesis of TN.

This study provides novel insights into the potential roles of BCL2A1 and DBT in trigeminal neuralgia, which have been rarely reported in previous research. Among them, BCL2A1 belongs to the BCL-2 protein family and is encoded by the BCL2A1 gene. Proteins in this family form heterologous or homodimeric structures and function as anti-apoptotic and pro-apoptotic regulators, participating in multiple cellular processes such as embryonic development, homeostasis, and tumorigenesis (Park et al., 2024; Vier et al., 2024). BCL2A1 is critical for maintaining immune system homeostasis. Studies demonstrate that during normal immune responses, upregulated BCL2A1 expression sustains the survival of activated T cells, ensuring effective immune reactions against pathogens. Following pathogen clearance, most activated T cells undergo apoptosis, accompanied by downregulated BCL2A1 expression to maintain immune cell population balance. Aberrant BCL2A1 expression may cause excessive T-cell apoptosis, leading to compromised immune function (Tuzlak et al., 2017). In diffuse large B-cell lymphoma (DLBCL), the anti-apoptotic protein BCL2A1 exhibits highly heterogeneous expression. Elevated BCL2A1 expression inhibits tumor cell apoptosis, enabling sustained proliferation and survival of malignant cells, where it plays a critical role in protecting cancer cells from death-inducing stimuli (Pieper et al., 2024). Therefore, BCL2A1 may play important roles in maintaining immune homeostasis and regulating tumor cell survival. These findings support the use of peripheral blood as a feasible and non-invasive material for biomarker screening in trigeminal neuralgia. Although peripheral blood cannot fully reflect the direct pathological damage of the TG, the detected alterations in immune and apoptosis-related molecules in blood can reflect the systemic inflammatory state involved in the progression of trigeminal neuralgia. Future validation of these biomarkers and related PCD mechanisms in trigeminal ganglion tissue is still needed to elucidate the connection between systemic immune dysregulation and localized trigeminal nerve damage.

Dihydrolipoamide branched-chain transacylase E2 is an intramitochondrial enzyme complex that takes part in the catabolic process of the branched-chain amino acids–namely isoleucine, leucine, and valine (Shi et al., 2025). This process occupies an important position in mitochondrial energy metabolism. Studies indicate that in the mucosal tissue of inflammatory bowel disease (IBD) patients, multiple inflammatory pathways are simultaneously activated. Dysfunctional DBT is associated with impaired branched-chain amino acid metabolism, which may affect immune cell proliferation, differentiation, and cytokine secretion, and is potentially linked to intestinal immune imbalance and the development of inflammatory bowel disease (Alayo et al., 2022). Maple Syrup Urine Disease (MSUD) is an autosomal recessive inherited disorder affecting the metabolism of branched-chain amino acids, which arises from impaired breakdown of these amino acids. The DBT gene encodes the E2 subunit of the branched-chain α-keto acid dehydrogenase complex (BCKDC) in the mitochondrial matrix. This subunit is critical to the oxidative metabolism process of branched-chain amino acids. Mutations in the DBT gene cause functional defects in BCKDC, which in turn results in the inability of branched-chain amino acids to undergo normal metabolism (Campanholi et al., 2021). At present, there is relatively little research on DBT and TN. We hypothesize that DBT functional deficiency may participate in neuroinflammation and immune imbalance processes by disrupting mitochondrial energy metabolism and immune cell function. Ultimately, this provides a novel perspective for understanding the specific roles of mitochondrial dysfunction and DBT/BCL2A1-mediated immune dysregulation in the development and progression of tumor necrosis encephalitis.

Gene Set Enrichment Analysis revealed that BCL2A1 and DBT exhibit co-enrichment in six key pathways (*P* < 0.05), including insulin signaling and Fcγ receptor-mediated phagocytosis. Additionally, within the ribosome pathway, 80 genes predominantly show negative correlations with both BCL2A1 and DBT. The insulin signaling pathway is critical for maintaining metabolic homeostasis. Insulin binding to its surface receptor (INSR) activates downstream signaling molecules, with the PI3K-Akt pathway serving as the core axis (Sharma et al., 2008). Notably, dysregulation of neuronal insulin signaling is linked to neurodegeneration (Arnold et al., 2018). The co-enrichment of BCL2A1 and DBT may disrupt the insulin signaling pathway, impairing energy metabolism in neurons. Fcγ receptor-mediated phagocytosis is primarily executed by immune cells (e.g., macrophages, neutrophils): antibody-coated pathogens or damaged cells are recognized by Fcγ receptors, triggering receptor cross-linking. This initiates signal cascades leading to cytoskeletal rearrangement and membrane remodeling, forming phagosomes that fuse with lysosomes for pathogen clearance (Daëron, 1997; Gordon, 2016). In TN patients with neuroinflammation, microglia may become aberrantly overactivated due to dysfunctional FcγR-mediated phagocytosis. BCL2A1 likely interferes with signal transduction, causing microglia to excessively phagocytose healthy neurons (Cao et al., 2010; Karavis et al., 2023). Studies have shown that ginsenoside Rg3 can boost Fc gamma receptor - dependent bacterial phagocytosis through the activation of the extracellular signal - regulated kinase 1/2 and p38 MAPK signaling pathways (Xin et al., 2019).

These results suggest that morphine administration elevates intracellular cAMP levels and activates protein kinase A, which in turn inhibits p38 MAPK. This inhibition further results in reduced actin polymerization, impaired FcgR-mediated phagocytosis, and diminished bacterial clearance (Ninković and Roy, 2012). Through the use of PD98059 (a specific inhibitor of MAPK/extracellular signal-regulated kinase kinase, or MAPK kinase), it was discovered that the activation of MAPK is indispensable for FcR-dependent activation of the nuclear factor NF-κB (Sánchez-Mejorada and Rosales, 1998).

BCL2A1 and DBT have different expression patterns and functions in TN, and their combination of targeted therapy may provide a more comprehensive intervention strategy. By regulating the expression or function of these genes, immune microenvironment, metabolic disorders, and energy metabolism can be improved simultaneously, thereby enhancing treatment efficacy. At present, most of the predicted drugs are remain unmarketed drugs, and the predicted therapeutic potential can be explored in combination with marketed homologous compounds. Future research can further explore the specific mechanisms of action of these genes and develop corresponding combination therapeutic drugs, providing new directions for the clinical management of TN.

The results of immune microenvironment correlation analysis suggest that biomarkers may influence disease progression and treatment response by affecting the activity and function of specific immune cells to modulate the immune microenvironment, thereby influencing disease progression and treatment response. Research on Langerhans cell histiocytosis (LCH) has revealed that circulating BRAFV600E+ peripheral blood mononuclear cells can infiltrate the brain, where monocyte-derived CD11a+ macrophages accumulate in large numbers. These cells overexpress senescence-related genes and multiple integrin subunits, disrupting the blood-brain barrier and triggering neuroinflammation and neurodegenerative pathologies (Wilk et al., 2023). In immunodeficiency diseases, patients with FMS-related tyrosine kinase 3 ligand (FLT3L) deficiency exhibit severe bone marrow hypoplasia, with one of the most notable phenotypes being severe monocytopenia. The reduction in monocytes compromises the body’s immune defense function (Momenilandi et al., 2024). In addition, regarding the second differentially infiltrated cell type, the identification of mesangial cells in peripheral blood must be interpreted with extreme caution. Mesangial cells are traditionally kidney-resident cells and are typically absent from systemic circulation (Cho et al., 2025; Choudhary et al., 2025). The detection of this signal in xCell deconvolution analysis likely reflects a technical artifact arising from transcriptomic cross-reactivity (Aran et al., 2017). The gene signatures used by the xCell algorithm may overlap between mesangial cells and other circulating cell populations, such as circulating mesenchymal stromal cells, which share similar fibroblast-like or contractile transcriptional characteristics (Ghazanfari et al., 2017). Alternatively, if this signal represents a genuine biological finding, it may reveal a novel “mesangial-like” mechanism in TN: circulating mesenchymal precursor cells could be mobilized by systemic neuroinflammation or vascular changes associated with trigeminal nerve compression. However, without validation by single-cell RNA sequencing or flow cytometry, this finding remains an intriguing but unconfirmed hypothesis.

This study comprehensively applied various bioinformatics methods to systematically explore the associations between TN, mitochondrial function, and 18 PCD-related genes. By constructing and validating nomograms for clinical prediction, analyzing transcription factors, predicting potential drugs, and assessing correlations between biomarkers and differential immune cell infiltration, provided a basis for personalized treatment. However, this study has several limitations: (1) This was a single-center exploratory study with only 10 pairs of trigeminal neuralgia patients and healthy controls, and the relatively limited sample size may affect statistical power and generalizability to some extent. (2) There are differences between peripheral blood characteristics and local pathological changes in the trigeminal nerve. The mitochondrial and programmed cell death-related markers screened in blood in this study may only reflect systemic immune-metabolic changes and cannot directly represent neuronal death. Follow-up studies using animal models are needed to verify whether the expression of BCL2A1 and DBT is synchronized between the trigeminal ganglion and peripheral blood. (3) The transcriptomic analysis in this study was based on peripheral whole blood rather than the TG, the core pathological site of trigeminal neuralgia. Although peripheral blood enables non-invasive and clinically translatable biomarker screening, it cannot fully represent the local molecular alterations in neural tissue. Future studies using clinical trigeminal ganglion specimens are warranted to validate these biomarkers and elucidate their roles in the local pathological mechanisms of trigeminal neuralgia. (4) This study identified BCL2A1 and DBT as biomarkers solely through bioinformatics approaches, without experimental validation using molecular biology techniques such as qPCR or Western Blot to confirm their differential expression at mRNA and protein levels in trigeminal neuralgia patients. The study conclusions therefore require further substantiation by experimental data. (5) This study only suggested potential associations between the screened biomarkers and mitochondrial function and PCD through bioinformatics analysis, without conducting functional experiments such as TUNEL staining or detection of molecular markers for ferroptosis, pyroptosis, and other PCD types. Therefore, the specific activation status of PCD types in trigeminal neuralgia could not be directly confirmed, and the related conclusions remain preliminary hypotheses awaiting further validation by functional experiments.

In follow-up studies, we will expand the sample size and conduct multi-center cohort validation, combined with clinical trigeminal ganglion specimens, molecular biology validation experiments, and cellular functional assays, to further clarify the regulatory mechanisms by which biomarkers mediate TN pathogenesis, thereby providing more substantial experimental support for subsequent disease mechanism research and the development of potential therapeutic targets.

## 5 Conclusion

In summary, this pilot study identified two potential biomarkers (BCL2A1 and DBT) associated with mitochondrial dysfunction and PCD in TN. Despite the constraints of the current sample size, these findings offer novel research orientations for the diagnosis and treatment of TN. Future large-scale studies are warranted to confirm their clinical utility.

## Data Availability

The datasets presented in this study can be found in online repositories. The names of the repository/repositories and accession number(s) can be found in the article/Supplementary material.

## Footnotes

1
https://www.omicstudio.cn/home

2
https://cran.r-project.org/package=ggVennDiagram

3
https://www.proteinatlas.org

4
http://www.genemania.org/

5
https://www.gsea-msigdb.org/gsea/msigdb

6
https://www.mirnet.ca/miRNet/home.xhtml

7
https://www.dgidb.org/

## Supplementary material

Supplementary Table 1 Gene expression levels in samples from the TN group and control group.

Supplementary Table 2 1,136 mitochondria-related genes and 1,548 programmed cell death-related genes.

Supplementary Table 3 Names of the 18 types of PCD and their corresponding gene data sources.

Supplementary Table 4 DEGs between TN and control groups.

Supplementary Table 5 GO and KEGG enrichment results of DEGs.

Supplementary Table 6 Result table of GSEA enrichment analysis for BCL2A1 and DBT.

Supplementary Figure 1 Nomogram constructed from the GSE186505 dataset. **(a)** Nomogram; **(b)** Calibration curve; **(c)** DCA curve.

## References

[B1] Alayo Q. A. Fenster M. Altayar O. Glassner K. L. Llano E. Clark-Snustad K. et al. (2022). Systematic review with meta-analysis: Safety and effectiveness of combining biologics and small molecules in inflammatory bowel disease. *Crohns Colitis 360* 4:otac002. 10.1093/crocol/otac002 35310082 PMC8924906

[B2] Andersen A. S. S. Heinskou T. B. Rochat P. Springborg J. B. Noory N. Smilkov E. A. et al. (2022). Microvascular decompression in trigeminal neuralgia - a prospective study of 115 patients. *J. Headache Pain* 23:145. 10.1186/s10194-022-01520-x 36402970 PMC9675260

[B3] Aran D. Hu Z. Butte A. J. (2017). xCell: Digitally portraying the tissue cellular heterogeneity landscape. *Genome Biol.* 18:220. 10.1186/s13059-017-1349-1 29141660 PMC5688663

[B4] Arnold S. E. Arvanitakis Z. Macauley-Rambach S. L. Koenig A. M. Wang H. Y. Ahima R. S. et al. (2018). Brain insulin resistance in type 2 diabetes and Alzheimer disease: Concepts and conundrums. *Nat. Rev. Neurol.* 14 168–181. 10.1038/nrneurol.2017.185 29377010 PMC6098968

[B5] Bezerra G. M. D. S. Leal P. R. L. Cavalcante-Neto J. F. Rivera A. da Ponte K. F. Cristino-Filho G. (2023). Microvascular decompression using autologous muscle graft for trigeminal neuralgia: A case series and meta-analysis. *Acta Neurochir.* 165 3833–3843. 10.1007/s00701-023-05871-5 38059995

[B6] Cai J. Yan Y. Zhang D. Zhu M. Wang Z. Zhang X. et al. (2020). Silencing of lncRNA Gm14461 alleviates pain in trigeminal neuralgia through inhibiting astrocyte activation. *IUBMB Life* 72 2663–2671. 10.1002/iub.2395 33141516

[B7] Campanholi D. R. R. Margutti A. V. B. Silva W. A. Garcia D. F. Molfetta G. A. Marques A. A. et al. (2021). Molecular basis of various forms of maple syrup urine disease in Chilean patients. *Mol. Genet. Genomic Med.* 9:e1616. 10.1002/mgg3.1616 33955723 PMC8172190

[B8] Cao S. Theodore S. Standaert D. G. (2010). Fcγ receptors are required for NF-κB signaling, microglial activation and dopaminergic neurodegeneration in an AAV-synuclein mouse model of Parkinson’s disease. *Mol. Neurodegener.* 5:42. 10.1186/1750-1326-5-42 20977765 PMC2975641

[B9] Chen H. Boutros P. C. (2011). VennDiagram: A package for the generation of highly-customizable Venn and Euler diagrams in R. *BMC Bioinformatics* 12:35. 10.1186/1471-2105-12-35 21269502 PMC3041657

[B10] Chen K. Wei X. Wang R. Yang L. Zou D. Wang Y. (2023). BMP7 alleviates trigeminal neuralgia by reducing oligodendrocyte apoptosis and demyelination. *J. Headache Pain* 24:143. 10.1186/s10194-023-01681-3 37875834 PMC10594892

[B11] Chen Y. Li X. Yang M. Liu S. B. (2024). Research progress on morphology and mechanism of programmed cell death. *Cell Death Dis.* 15:327. 10.1038/s41419-024-06712-8 38729953 PMC11087523

[B12] Cho J. M. Park S. J. Kim Y. J. Lee S. Lee S. Im D. W. et al. (2025). Soluble ST2 is an early marker and treatment target for hypertensive nephrosclerosis signatured in glomerular mesangial cells. *Transl. Res.* 279 16–26. 10.1016/j.trsl.2025.03.001 40096886

[B13] Choudhary S. Picut C. Vargas S. R. Otis D. Coskran T. M. Karanian D. et al. (2025). Mesangial cell hypercellularity and iron accumulation in the kidney associated with administration of a sickle hemoglobin modulator in CD-1 mice. *Vet. Pathol.* 62 397–407. 10.1177/03009858241306400 39711519

[B14] Cruccu G. Finnerup N. B. Jensen T. S. Scholz J. Sindou M. Svensson P. et al. (2016). Trigeminal neuralgia: New classification and diagnostic grading for practice and research. *Neurology* 87 220–228. 10.1212/WNL.0000000000002840 27306631 PMC4940067

[B15] Daëron M. (1997). Fc receptor biology. *Annu. Rev. Immunol.* 15 203–234. 10.1146/annurev.immunol.15.1.203 9143687

[B16] Danial N. N. Korsmeyer S. J. (2004). Cell death: Critical control points. *Cell* 116 205–219. 10.1016/s0092-8674(04)00046-7 14744432

[B17] De Stefano G. Litewczuk D. Mollica C. Di Pietro G. Galosi E. Leone C. et al. (2023). Sex differences in trigeminal neuralgia: A focus on radiological and clinical characteristics. *Neurol. Sci.* 44 4465–4472. 10.1007/s10072-023-06923-5 37436558 PMC10641090

[B18] Friedman J. Hastie T. Tibshirani R. (2010). Regularization paths for generalized linear models via coordinate descent. *J. Stat. Softw.* 33 1–22. 10.18637/jss.v033.i01 20808728 PMC2929880

[B19] Ghazanfari R. Zacharaki D. Li H. Ching Lim H. Soneji S. Scheding S. (2017). Human primary bone marrow mesenchymal stromal cells and their in vitro progenies display distinct transcriptional profile signatures. *Sci. Rep.* 7:10338. 10.1038/s41598-017-09449-x 28871088 PMC5583257

[B20] Gibellini L. Moro L. (2021). Programmed cell death in health and disease. *Cells* 10:1765. 10.3390/cells10071765 34359935 PMC8303776

[B21] Glick D. Barth S. Macleod K. F. (2010). Autophagy: Cellular and molecular mechanisms. *J. Pathol.* 221 3–12. 10.1002/path.2697 20225336 PMC2990190

[B22] Gordon S. (2016). Phagocytosis: An immunobiologic process. *Immunity* 44 463–475. 10.1016/j.immuni.2016.02.026 26982354

[B23] Green D. Kroemer G. (1998). The central executioners of apoptosis: Caspases or mitochondria? *Trends Cell Biol.* 8 267–271. 10.1016/s0962-8924(98)01273-2 9714597

[B24] Gu Z. Hübschmann D. (2022). Make interactive complex heatmaps in R. *Bioinformatics* 38 1460–1462. 10.1093/bioinformatics/btab806 34864868 PMC8826183

[B25] Guan X. Y. Dong X. Wang Y. X. Xu B. C. Wu X. B. (2025). Mitochondrial dysfunction in trigeminal ganglion contributes to nociceptive behavior in a nitroglycerin-induced migraine mouse model. *Mol. Pain* 21:17448069251332100. 10.1177/17448069251332100 40110756 PMC12035203

[B26] Gustavsson E. K. Zhang D. Reynolds R. H. Garcia-Ruiz S. Ryten M. (2022). ggtranscript: An R package for the visualization and interpretation of transcript isoforms using ggplot2. *Bioinformatics* 38 3844–3846. 10.1093/bioinformatics/btac409 35751589 PMC9344834

[B27] Headache Classification Committee of the International Headache Society [IHS] (2013). The international classification of headache disorders, 3rd edition (beta version). *Cephalalgia* 33 629–808. 10.1177/0333102413485658 23771276

[B28] Hu X. Wang J. Ju Y. Zhang X. Qimanguli W. Li C. et al. (2022). Combining metabolome and clinical indicators with machine learning provides some promising diagnostic markers to precisely detect smear-positive/negative pulmonary tuberculosis. *BMC Infect. Dis.* 22:707. 10.1186/s12879-022-07694-8 36008772 PMC9403968

[B29] Karavis M. Y. Siafaka I. Vadalouca A. Georgoudis G. (2023). Role of microglia in neuropathic pain. *Cureus* 15:e43555. 10.7759/cureus.43555 37719474 PMC10503876

[B30] Li J. Chen X. Li X. Hu R. Yao W. Mei W. et al. (2020). Upregulation of Cdh1 in the trigeminal spinal subnucleus caudalis attenuates trigeminal neuropathic pain via inhibiting GABAergic neuronal apoptosis. *Neurochem. Int.* 133:104613. 10.1016/j.neuint.2019.104613 31785347

[B31] Liu P. Xu H. Shi Y. Deng L. Chen X. (2020). Potential molecular mechanisms of plantain in the treatment of gout and hyperuricemia based on network pharmacology. *Evid. Based Complement. Alternat. Med.* 2020:3023127. 10.1155/2020/3023127 33149752 PMC7603577

[B32] Lopez M. Spehner L. André F. Viot J. Seffar E. Marguier A. et al. (2025). Exploring the role of ESR1 mutations in metastatic hormone receptor-positive breast cancer T cell immune surveillance disruption. *Breast Cancer Res.* 27:19. 10.1186/s13058-025-01962-6 39920833 PMC11806781

[B33] Melek L. N. Smith J. G. Karamat A. Renton T. (2019). Comparison of the neuropathic pain symptoms and psychosocial impacts of trigeminal neuralgia and painful posttraumatic trigeminal neuropathy. *J. Oral Facial Pain Headache* 33 77–88. 10.11607/ofph.2157 30703173

[B34] Menzies F. M. Fleming A. Caricasole A. Bento C. F. Andrews S. P. Ashkenazi A. et al. (2017). Autophagy and neurodegeneration: Pathogenic mechanisms and therapeutic opportunities. *Neuron* 93 1015–1034. 10.1016/j.neuron.2017.01.022 28279350

[B35] Miao R. Jiang C. Chang W. Y. Zhang H. An J. Ho F. et al. (2023). Gasdermin D permeabilization of mitochondrial inner and outer membranes accelerates and enhances pyroptosis. *Immunity* 56 2523–2541.e8. 10.1016/j.immuni.2023.10.004 37924812 PMC10872579

[B36] Momenilandi M. Lévy R. Sobrino S. Li J. Lagresle-Peyrou C. Esmaeilzadeh H. et al. (2024). FLT3L governs the development of partially overlapping hematopoietic lineages in humans and mice. *Cell* 187 2817–2837.e31. 10.1016/j.cell.2024.04.009 38701783 PMC11149630

[B37] Ninković J. Roy S. (2012). Morphine decreases bacterial phagocytosis by inhibiting actin polymerization through cAMP-, Rac- 1-, and p38 MAPK-dependent mechanisms. *Am. J. Pathol.* 180 1068–1079. 10.1016/j.ajpath.2011.11.034 22248582 PMC3349892

[B38] Park C. W. Lee E. M. Shin S. H. Lee C. Won J. K. (2024). The intensity of BCL2A1 expression increases according to the stage progression of acute histologic chorioamnionitis in the extra-placental membranes of spontaneous preterm birth. *Life* 14:1535. 10.3390/life14121535 39768244 PMC11677416

[B39] Pertea M. Kim D. Pertea G. M. Leek J. T. Salzberg S. L. (2016). Transcript-level expression analysis of RNA-seq experiments with HISAT, StringTie and Ballgown. *Nat. Protoc.* 11 1650–1667. 10.1038/nprot.2016.095 27560171 PMC5032908

[B40] Pieper N. M. Schnell J. Bruecher D. Knapp S. Vogler M. (2024). Inhibition of bromodomain and extra-terminal proteins targets constitutively active NFκB and STAT signaling in lymphoma and influences the expression of the antiapoptotic proteins BCL2A1 and c-MYC. *Cell Commun. Signal.* 22:415. 10.1186/s12964-024-01782-9 39192247 PMC11348570

[B41] Qin H. Abulaiti A. Maimaiti A. Abulaiti Z. Fan G. Aili Y. et al. (2023). Integrated machine learning survival framework develops a prognostic model based on inter-crosstalk definition of mitochondrial function and cell death patterns in a large multicenter cohort for lower-grade glioma. *J. Transl. Med.* 21:588. 10.1186/s12967-023-04468-x 37660060 PMC10474752

[B42] Reuter S. (2022). Mitochondria - More than just batteries for cellular function. *Acta Physiol.* 235:e13852. 10.1111/apha.13852 35723182

[B43] Sánchez-Mejorada G. Rosales C. (1998). Fcgamma receptor-mediated mitogen-activated protein kinase activation in monocytes is independent of Ras. *J. Biol. Chem.* 273 27610–27619. 10.1074/jbc.273.42.27610 9765295

[B44] Shankar Kikkeri N. Nagalli S. (2025). *Trigeminal Neuralgia.* Treasure Island, FL: StatPearls. 32119373

[B45] Shannon P. Markiel A. Ozier O. Baliga N. S. Wang J. T. Ramage D. et al. (2003). Cytoscape: A software environment for integrated models of biomolecular interaction networks. *Genome Res.* 13 2498–2504. 10.1101/gr.1239303 14597658 PMC403769

[B46] Sharma M. D. Garber A. J. Farmer J. A. (2008). Role of insulin signaling in maintaining energy homeostasis. *Endocr. Pract.* 14 373–380. 10.4158/EP.14.3.373 18463047

[B47] Shi J. Wu Q. Sang M. Mao L. (2025). Common regulatory mechanisms mediated by cuproptosis genes in inflammatory bowel disease and major depressive disorder. *Genes* 16:339. 10.3390/genes16030339 40149491 PMC11942124

[B48] Svedung Wettervik T. Snel D. Kristiansson P. Ericson H. Abu Hamdeh S. (2023). Incidence of trigeminal neuralgia: A population-based study in Central Sweden. *Eur. J. Pain* 27 580–587. 10.1002/ejp.2081 36680398

[B49] Tuzlak S. Schenk R. L. Vasanthakumar A. Preston S. P. Haschka M. D. Zotos D. et al. (2017). The BCL-2 pro-survival protein A1 is dispensable for T cell homeostasis on viral infection. *Cell Death Differ.* 24 523–533. 10.1038/cdd.2016.155 28085151 PMC5344212

[B50] Vakifahmetoglu-Norberg H. Ouchida A. T. Norberg E. (2017). The role of mitochondria in metabolism and cell death. *Biochem. Biophys. Res. Commun.* 482 426–431. 10.1016/j.bbrc.2016.11.088 28212726

[B51] Vier J. Häcker G. Kirschnek S. (2024). Contribution of A1 to macrophage survival in cooperation with MCL-1 and BCL-XL in a murine cell model of myeloid differentiation. *Cell Death Dis.* 15:677. 10.1038/s41419-024-07064-z 39285161 PMC11405755

[B52] Wang L. Long M. Wang M. Peng S. Chen G. Zhou J. et al. (2020). Trigeminal neuralgia causes neurodegeneration in rats associated with upregulation of the CD95/CD95L pathway. *Mol. Pain* 16:1744806920908092. 10.1177/1744806920908092 32013712 PMC7054737

[B53] Wang L. Wang D. Yang L. Zeng X. Zhang Q. Liu G. et al. (2022). Cuproptosis related genes associated with Jab1 shapes tumor microenvironment and pharmacological profile in nasopharyngeal carcinoma. *Front. Immunol.* 13:989286. 10.3389/fimmu.2022.989286 36618352 PMC9816571

[B54] Wilk C. M. Cathomas F. Török O. Le Berichel J. Park M. D. Bigenwald C. et al. (2023). Circulating senescent myeloid cells infiltrate the brain and cause neurodegeneration in histiocytic disorders. *Immunity* 56 2790–2802.e6. 10.1016/j.immuni.2023.11.011 38091952 PMC11587932

[B55] Wu T. Hu E. Xu S. Chen M. Guo P. Dai Z. et al. (2021). clusterProfiler 4.0: A universal enrichment tool for interpreting omics data. *Innovation* 2:100141. 10.1016/j.xinn.2021.100141 34557778 PMC8454663

[B56] Wu T. H. Hu L. Y. Lu T. Chen P. M. Chen H. J. Shen C. C. et al. (2015). Risk of psychiatric disorders following trigeminal neuralgia: A nationwide population-based retrospective cohort study. *J. Headache Pain* 16:64. 10.1186/s10194-015-0548-y 26174508 PMC4501948

[B57] Xin C. Kim J. Quan H. Yin M. Jeong S. Choi J. I. et al. (2019). Ginsenoside Rg3 promotes Fc gamma receptor-mediated phagocytosis of bacteria by macrophages via an extracellular signal-regulated kinase 1/2 and p38 mitogen-activated protein kinase-dependent mechanism. *Int. Immunopharmacol.* 77:105945. 10.1016/j.intimp.2019.105945 31644962

[B58] Yang J. Xie S. Guo J. Zhou Y. Yang Y. Sun Z. et al. (2025). Restoration of mitochondrial function alleviates trigeminal neuropathic pain in mice. *Free Radic. Biol. Med.* 226 185–198. 10.1016/j.freeradbiomed.2024.11.011 39528053

[B59] Yuan J. Yankner B. A. (2000). Apoptosis in the nervous system. *Nature* 407 802–809. 10.1038/35037739 11048732

[B60] Zakrzewska J. M. Wu J. Mon-Williams M. Phillips N. Pavitt S. H. (2017). Evaluating the impact of trigeminal neuralgia. *Pain* 158 1166–1174. 10.1097/j.pain.0000000000000853 28114183

[B61] Zhao W. Fang H. Wang T. Yao C. (2024). Identification of mitochondria-related biomarkers in childhood allergic asthma. *BMC Med. Genomics* 17:141. 10.1186/s12920-024-01901-y 38783263 PMC11112767

[B62] Zou Y. Xie J. Zheng S. Liu W. Tang Y. Tian W. et al. (2022). Leveraging diverse cell-death patterns to predict the prognosis and drug sensitivity of triple-negative breast cancer patients after surgery. *Int. J. Surg.* 107:106936. 10.1016/j.ijsu.2022.106936 36341760

