# Correction: Identification and validation of candidate biomarkers associated with mitochondria and 18 types of programmed cell death in trigeminal neuralgia: insights from transcriptome sequencing

**DOI:** 10.3389/fnmol.2026.1932955

**Published:** 2026-08-11

**Authors:** Liqin Gao, Hongchen Shi, Lulu Xi, Xiaohui Liu, Yue Ren, Qiujun Wang

**Affiliations:** 1 Department of Pain, The Second Hospital of Hebei Medical University, Shijiazhuang, China; 2 Department of Anesthesiology, Hebei Medical University Third Hospital, Shijiazhuang, China

**Keywords:** machine learning, mitochondria, nomogram, programmed cell death, trigeminal neuralgia

In the published article, in section “*3.1 Overview of transcriptome profiles in TN*,” paragraph 2, “ENSG00000279249” appears in error and should not have been included. Instead, “ENSG00000279249” should be corrected to “ENSG00000270249.” The corrected paragraph appears below.

“After performing differential expression analysis between TN and controls in RNA-seq data, a total of 1,369 DEGs were determined, along with 703 down-regulated and 666 upregulated genes in disease category (*P* < 0.05; Figures 1c, d and Supplementary Table 4). Among them, ENSG00000259171, ENSG00000270249, and ENSG00000173366 were ENSG numbers and cannot be converted into gene symbols. These genes were considered to be newly discovered in the current database version, or their gene symbols had not been determined yet.”

The original version of this article has been updated.

