# Supplementary figures and images for: Identification and validation of candidate biomarkers associated with mitochondria and 18 types of programmed cell death in trigeminal neuralgia: insights from transcriptome sequencing
Source: Front Mol Neurosci. 2026 Jun 24;19:1798469. doi: 10.3389/fnmol.2026.1798469 (PMC13341618; doi:10.3389/fnmol.2026.1798469)

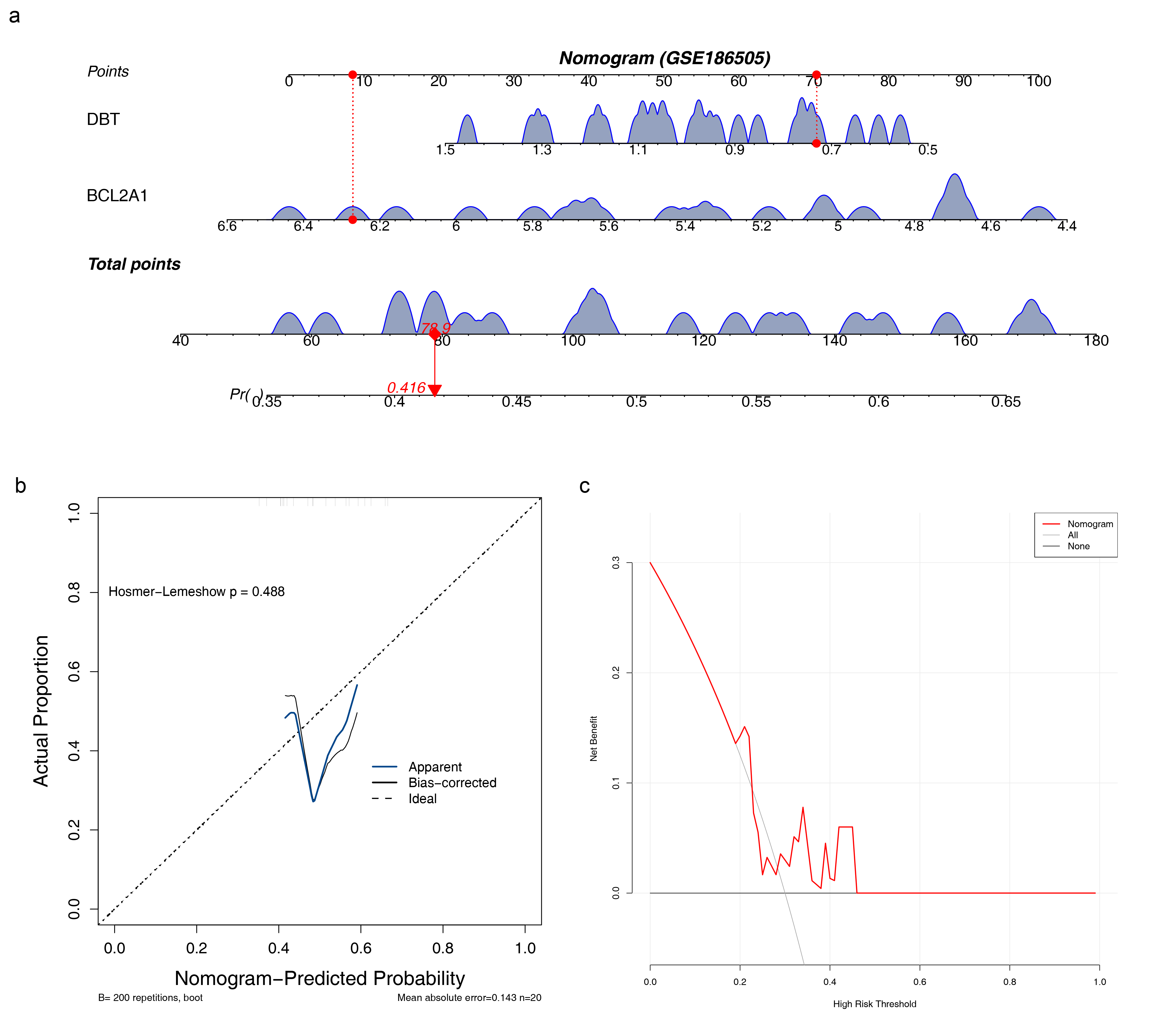

Supplement: Supplementary Table 1 — Gene expression levels in samples from the TN group and control group. [file Data_Sheet_1.zip › Supplementary Figure 1.tif]
